# Resistance Isn't Futile: The Physiological Basis of the Health Effects of Resistance Exercise in Individuals With Type 1 Diabetes

**DOI:** 10.3389/fendo.2019.00507

**Published:** 2019-08-02

**Authors:** Olivia McCarthy, Othmar Moser, Max L. Eckstein, Rachel Deere, Steve C. Bain, Jason Pitt, Richard M. Bracken

**Affiliations:** ^1^Applied Sport, Technology, Exercise and Medicine Research Centre (A-STEM), College of Engineering, Swansea University, Swansea, United Kingdom; ^2^Diabetes Research Group, Medical School, Swansea University, Swansea, United Kingdom; ^3^Cardiovascular Diabetology Research Group, Division of Endocrinology and Diabetology, Department of Internal Medicine, Medical University of Graz, Graz, Austria

**Keywords:** resistance exercise, type 1 diabetes, physical activity, strength training, weights

## Abstract

The importance of regular exercise for glucose management in individuals with type 1 diabetes is magnified by its acknowledgment as a key adjunct to insulin therapy by several governmental, charitable, and healthcare organisations. However, although actively encouraged, exercise participation rates remain low, with glycaemic disturbances and poor cardiorespiratory fitness cited as barriers to long-term involvement. These fears are perhaps exacerbated by uncertainty in how different forms of exercise can considerably alter several acute and chronic physiological outcomes in those with type 1 diabetes. Thus, understanding the bodily responses to specific forms of exercise is important for the provision of practical guidelines that aim to overcome these exercise barriers. Currently, the majority of existing exercise research in type 1 diabetes has focused on moderate intensity continuous protocols with less work exploring predominately non-oxidative exercise modalities like resistance exercise. This is surprising, considering the known neuro-muscular, osteopathic, metabolic, and vascular benefits associated with resistance exercise in the wider population. Considering that individuals with type 1 diabetes have an elevated susceptibility for complications within these physiological systems, the wider health benefits associated with resistance exercise may help alleviate the prevalence and/or magnitude of pathological manifestation in this population group. This review outlines the health benefits of resistance exercise with reference to evidence in aiding some of the common complications associated with individuals with type 1 diabetes.

## Introduction

Notwithstanding their etiological, pathophysiological, and epidemiological complexities, the severity and incidence of complications associated with type 1 diabetes (T1D) are lower in physically active individuals (1, 2). Conversely, at a population level, sedentariness constitutes the fourth leading risk factor for mortality (3), such that individuals with the highest rates of sedentariness are up three times more likely to suffer premature mortality than their physically active counterparts (4). Therefore, whilst pharmaceutical and technological therapies aid glycaemic management, the utility of simple, holistic approaches such as regular physical activity (PA) constitute attractive adjunct treatment options, that alongside standardised medical care facilitate the improvement of T1D related pathological complications. However, a research and advocatory emphasis has been placed on the implementation of sustainable moderate intensity continuous exercise (MICE) in those with T1D, with less discernible evidence exploring alternative exercise modalities such as resistance exercise (RE). Whilst the health benefits associated with MICE are by no means immaterial, longstanding T1D is often accompanied by complications that impact the ability of participants to comfortably perform their rhythmic and/or pounding nature. Furthermore, MICE often fails to maximise the development of skeletal muscle mass and strength, both of which are features that facilitate improvements in various metabolic, neuroendocrine, and osteopathic processes.

As such, the utility of incorporating RE to assist the functional development of skeletal muscle integrity constitutes an important feature of the current exercise guidelines for those with T1D (5).

## Methods

The authors undertook a detailed PubMed literature search for the following key words: “resistance exercise,” “exercise,” “physical activity,” “strength training,” “weight training,” “type 1 diabetes,” “T1DM.” The reference lists of systematic reviews, reviews, and included as well as excluded articles were manually screened for studies considered to be relevant. The literature search was conducted by the corresponding and secondary lead authors with additional suggestions and/or missed literature being provided by co-authors when necessary.

## Importance of Physical Activity and Exercise in People With Type 1 Diabetes

Research has consistently demonstrated the inverse relationship between PA and a reduced risk of diabetes-related complications (6, 7), including both mental well-being (8), life-expectancy (4, 7), and health related quality of life (9). In addition to the advocation of frequent PA, there is considerable evidence supporting the inclusion of regular exercise as an adjunct therapeutic strategy in people with T1D (10). Definitively, exercise describes activities completed with a structured and intentional approach to maintaining or attaining improvements in physical fitness (11). The increased metabolic activity of skeletal muscle during exercise evokes an adaptive response of several integrative processes, including the cardio-respiratory, vascular, and metabolic systems. As a reflection of the integrative functions of these physiological systems, cardio-respiratory fitness (CRF) is often used as a barometer for physical fitness and reflects the ability of the circulatory-and-respiratory systems to supply the muscular system with the increasing oxygen (O_2_) demand experienced during sustained PA. Although the topic remains controversial, lower CRF has been reported in individuals with-vs.-without T1D (12). However, this finding appears to be closely related to glycaemic control, which can considerably influence acute exercise tolerance (12–14). Whilst considerable shifts in both cellular and tissue homeostasis are observed following most exercise activities, these processes are magnified following higher-intensity exercise, which represents a potent physiological stress. In the long-term, this acute stressor functions to optimise the body for subsequent subjection to bouts of physiological or indeed pathophysiological insults. Therefore, the type and intensity of the exercise stimulus can considerably influence the acute physiological and chronic adaptive responses that occur.

## Exercise Definition and Classification

**Figure 1:**
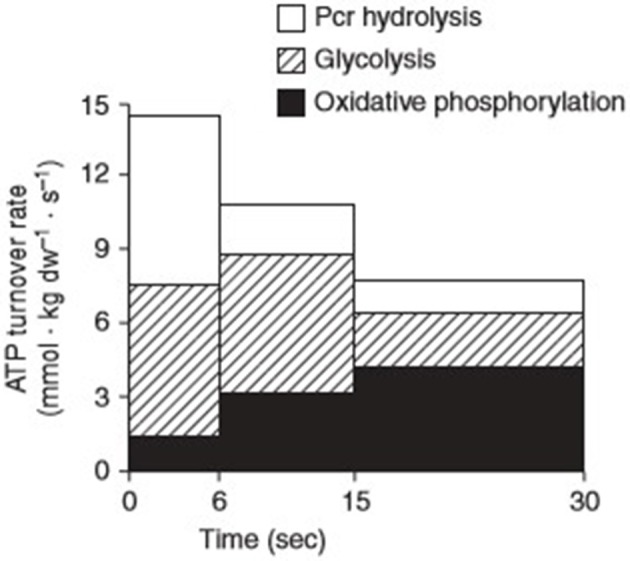
Contributions of the three major energy pathways; phosphocreatine (Pcr), glycolysis, and oxidative phosphorylation to adenosine triphosphate (ATP) turnover during 30 s of maximal isokinetic cycling exercise. Reprinted from Parolin et al. [(15), Figure 7B]. Copyright ©1999 the American Physiological Society.

The word “exercise” is often used interchangeably and encompasses several adjustable variables including modality, frequency, intensity, and duration. Metabolically speaking, the energy contribution to a given task is determined by the intensity of effort required for its performance. These metabolic disturbances activate several kinases and phosphatases, necessary not only for the immediate supply of energy to sustain contractional output, but also the synthesis of genetic transcriptions that produce an adaptive phenotype for subsequent functional demands. Generally, the pathways responsible for the provision of adenosine triphosphate (ATP) to skeletal muscle are categorised as aerobic i.e., with the use of O_2_ or anaerobic i.e., without the use of O_2_. In each pathway, muscle must convert free adenosine diphosphate (ADP) and inorganic phosphate (Pi) to ATP. The regenerated ATP is then made available to the myosin ATPase enzymes that facilitate the contractile processes that enable movement. During low to moderate intensity exercise, the rate of O_2_ supply necessary for continued contraction is comfortably met via the oxidative metabolism of carbohydrates and lipids. During intense physical exercise conditions of cellular hypoxia are experienced, as the rate of O_2_ demand far exceeds the rate of supply capable via oxidative phosphorylation. Under such circumstances, anaerobic metabolism becomes the predominate means via which ATP is produced. The process involves the degradation of phosphocreatine (PCr) i.e., the ATP-CP system and the production of lactate within the glycolytic pathway i.e., the glycolytic system (Figure 1).

Essentially, a continuum exists that enables physical exercise activities to be grouped into low, moderate, and high intensity efforts. However, it should be noted that no exercise is ever considered “exclusively” aerobic or anaerobic, rather both pathways are activated simultaneously with one predominating fuel provision. Nonetheless, the terms provide reference guides that can be used generally to classify exercise intensities. Conventionally speaking, predominately aerobic exercise imposes a high-frequency (repetition), low power output (load) demand on muscular contraction. These characteristics are often observed during MICE including endurance orientated activities. Conversely, predominately anaerobic exercise mandates a low-frequency, high-resistance power output including both high intensity exercise (HIIE) and RE.

### Resistance Exercise Definition

Resistance exercise or “strength/weight” exercise is a term used to describe exercise that mandates the body's musculature to move against an opposing force, addressing the functional behaviours mandatory to human movement. Resistance exercise training (RET) is the process of developing strength, flexibility, coordination, and stamina through performing exercises (at self-selected, high-intensity efforts) that involve multiple joints and muscle groups. RE be implemented using a multitude of training modalities, including isokinetic resistance, variable resistance, isometric resistance, and explosive plyometrics. This is important, as the term “resistance exercise” is often strictly associated with the use of equipment-based/external loads placed on the body in a strength orientated fashion. However, it actually reflects the exercise continuum—from which program design allows variable adaptation. Fundamentally the acute program variables (muscle action, intensity and volume, exercise selection and order, rest interval, repetition velocity, and frequency) dictate the specific training outcome (i.e., muscular strength, hypertrophy, maximal strength, or power). As such, there appears to be a specific relationship between the training stimulus and the adaptive response. Due to its malleability RE can be easily adapted to suit the attainment of a specific exercise goal. Indeed, small adjustments in the load, velocity and duration of a typical RE movement can substantially alter the physiological response that occurs.

### Resistance Exercise Guidelines

As per the latest position statement by the American Diabetes Association (ADA), the adoption and maintenance of PA are critical foci for blood glucose management and overall health maintenance in individuals with T1D (5). Indeed, as part of their core guidelines, the ADA state that individuals with diabetes should engage in 150 min or more of moderate-to-vigorous intensity activity weekly. Guidelines suggest spreading these minutes over 3 days per week, with no more than 2 consecutive days passing without activity. Shorter durations (minimum 75 min per week) of vigorous-intensity or interval sprint training may be sufficient for younger and more physically fit individuals. Furthermore, the specific advocation of RE is included, and details that adults with T1D should engage in 2–3 sessions per week on non-consecutive days. Encouragingly, this also aligns with the latest position stand by the American College of Sports Medicine, who advocate the implementation of RE for each of the major muscle groups on ≥2 days per week in healthy adults (11). Finally, considering the elevated susceptibility to diabetes related complications, the utility, safety and physiological adaptations associated with RE are increasingly important. Due to its ability to train the peripheral muscles without producing extensive cardiovascular stress, dynamic interval based RE, using loads of 50–60% 1-repetition maximum (1RM), has been established as a safe and effective mode of exercise in high cardiovascular disease (CVD) risk cohorts (16).

Despite these advocatory guidelines, there is currently a lack research comprehensively detailing the physiological responses to RE in individuals with T1D (Table 1). Moreover, in what little RE-focused research pertinent to T1D that does exist, there is often a lack of program variability, which may overlook important responses to alterations in acute program variables i.e., time under tension, repetition ranges and exercise selection. Furthermore, existing research has mostly explored acute sessions, with participant numbers failing to exceed 15. Finally, the cohorts used have been relatively homogenous, made up of predominately young, relatively healthy, Caucasian male participants. This not only makes wider generalisation difficult, but also overlooks important gender, ethnic and age-related variables.

**Table 1:** Synopsis of the resistance exercise interventions in human participants with type 1 diabetes.

**References**	**Title**	**Participant characteristics**	**Exercise characteristics**	**Main outcome**
Durak et al. (17)	Randomized crossover study of effect of resistance training on glycaemic control, muscular strength, and cholesterol in type I diabetic men	***N*** = Eight adults **Age (years):** 31 ± 3.5 **BMI (kg/m**^**2**^**):** 25.9 ± 0.48 **Gender: ♂** 8 ♀ 0 **VO**_**2max**_ **(ml·kg·min**^**−1**^**):** **HbA1c (%):** **Diabetes duration:** 12.3 ± 9.8	**Intervention:** Chronic **Intervention duration:** 10 weeks. 3* p/w. **Exercise duration**: 60 min **Exercise Modality**: Whole body RE 6 upper body and 4 lower body exercises Major to minor muscle group sequence **Exercise Intensity**: Heavy, progressive RE designed to reach muscular failure in the latter sets. <12 reps per exercise. Total of 40–50 sets during a typical session. Rests between sets ranged from 30 s to 2 min	Glycaemia
Jimenez et al. (18)	Insulin-sensitivity response to a single bout of resistive exercise in type 1 diabetes mellitus	***N*** = Fourteen adults TG: 7 CG: 7 **Age (years):** TG: 23.4 ± 4.0 CG: 26.3 ± 6.7 **BMI (kg/m**^**2**^**):** TG: 28.3 CG: 24.2 **Gender:** ♂ 11 ♀ 3 **Co-morbidities:** **-Exercise**: Physically active **VO**_**2max**_ **(ml·kg·min**^**−1**^**):-** **HbA1c (%):** 6.8 ± 1.5 **Diabetes duration: -**	**Intervention:** Acute **Exercise duration**: **Exercise Modality**: Lower limb **Exercise Intensity**: 80% 5 sets 6 repetitions **Exercise Classification:** Strength	Glycaemia, CVD biomarkers
D'hooge et al. (19)	Influence of combined aerobic and resistance training on metabolic control, cardiovascular fitness and quality of life in adolescents with type 1 diabetes: a randomized controlled trial	***N*** = Sixteen Children **Age (years):** 10–18 **BMI (kg/m**^**2**^**):** **Gender:** **VO**_**2max**_ **(ml·kg·min**^**−1**^**):** **HbA1c (%):** **Diabetes duration:**	**Intervention:** Chronic **Intervention duration:** 20 weeks 2* p/w **Exercise duration**: 70 min **Exercise Modality**: Combined AE and RE **Exercise Intensity**: RE; - 20 RM 0–6 weeks - 17 RM 6–12 weeks - 12 RM 12–20 weeks	Glycaemia, CRF, Quality of Life
Yardley et al. (20) Yardley et al. (21)	Performing resistance exercise before vs. after aerobic exercise influences growth hormone secretion in type 1 diabetes Effects of performing resistance exercise before vs. after aerobic exercise on glycemia in type 1 diabetes	***N*** = Twelve adults **Age (years):** 31.8 ± 15.3 **BMI (kg/m**^**2**^**):** 25.3 ± 3.0 **Gender:** Mixed **♂** 10 ♀ 2 **VO**_**2max**_ **(ml·kg·min**^**−1**^**)** 51.2 ± 10.8 **HbA1c (%):**7.1 ± 1.1 **Diabetes duration:** 12.5 ± 10.0	**Intervention:** Acute **Exercise duration**: 45 min **Exercise Modality**: AE before RE (AR) RE before AE (RA) **Exercise Intensity**: AE 60% VO_2peak_ RE: 67% 1RM. 3 sets 10 reps **RE Classification:** Hypertrophy	Glycaemia
Silveira et al. (22)	Acute effects of different intensities of resistance training on glycaemic fluctuations in patients with type 1 diabetes mellitus	***N*** = Twelve adults **Age (years):** 24.4 ± 6.4 **BMI (kg/m**^**2**^**): 21.3 ± 2.9** **Gender:** ♂ 6 ♀ 6 **VO**_**2max**_ **(ml·kg·min**^**−1**^**):** **HbA1c (%):** **Diabetes duration: 7.3 ± 6.8**	**Intervention:** Acute **Exercise duration**: 70 min **Exercise Modality**: RE **Exercise Intensity**: 60% 1RM vs. 89% 1RM	Glycaemia
Turner et al. (23)	Reductions in resistance exercise-induced hyperglycaemic episodes are associated with circulating interleukin-6 in Type 1 diabetes	***N*** = Eight adults **Age (years):** 38 **BMI (kg/m**^**2**^**):** 26.9 ± 4.1 **Gender:** Mixed **♂** 7 ♀ 1 **VO**_**2max**_ **(ml·kg·min**^**−1**^**):** **HbA1c (%):** 8.7 ±1.0 **Diabetes duration (years):** 15 ±13	**Intervention:** Acute **Exercise duration**: 42 min **Exercise Modality**: Circuit RE Eight whole body exercises **Exercise Intensity**: RE: 67% 1RM. 3 sets 10 reps **RE Classification:** Hypertrophy	Glycaemia and Inflammation
Turner et al. (24)	Impact of single and multiple sets of resistance exercise in type 1 diabetes	***N*** = Eight adults **Age (years):** 38 **BMI (kg/m**^**2**^**):** 26.9 ± 4.1 **Gender:** Mixed **♂** 7 ♀ 1 **VO**_**2max**_ **(ml·kg·min**^**−1**^**):** **HbA1c (%):** 8.7 ±1.0 **Diabetes duration (years):** 15 ±13	**Intervention:** Acute **Exercise duration**: 42 min Set 1 (14 min) Set 2 (28 min) Set 3 (42 min) **Exercise Modality**: Circuit RE Eight whole body exercises **Exercise Intensity:** RE: 67% 1RM. 3 sets 10 reps **RE Classification:** Hypertrophy	Glycaemia
Waclawovsky et al. (25)	Exercise on progenitor cells in healthy subjects and patients with type 1 diabetes	***N*** = Fourteen adults **Age (years):** 30.3 ± 1.6 **BMI (kg/m**^**2**^**):** 26.2 ± 0.8 **Gender: ♂** 14 **VO**_**2peak**_ **(ml·kg·min**^**−1**^**):** 37.1 ± 1.4 **HbA1c (%):**7.7% ± 0.2 **Diabetes duration (years):** 30 ± 1.6	**Intervention:** Acute **Exercise duration:** 40 min **Exercise Modality:** Combined AE and RE AE (cycle ergometry) RE (four lower limb exercises) **Exercise Intensity:** AE: 40–60% VO_2max_ RE: 60% 1RM **RE Classification:** Hypertrophy	Microvascular
Turner et al. (26)	Similar magnitude of post-exercise hyperglycaemia despite manipulating resistance exercise intensity in type 1 diabetes individuals	***N*** = Eight adults **Age (years):** 34 ± 7 **BMI (kg/m**^**2**^**):** 25.7 ± 1.6 **Gender:** Mixed **♂** 7 ♀ 1 **VO**_**2max**_ **(ml·kg·min**^**−1**^**):** **HbA1c (%):** 8.7 ±1.0 **Diabetes duration (years):** 15 ±13	**Intervention:** Acute **Exercise duration:** 42 min **Exercise Modality**: Circuit RE Six whole body exercises **Exercise Intensity:** 2 sets of 20 reps @ 30% 1RM (LOW) 2 sets of 10 reps @ 60% 1RM (MOD) **RE Classification:** Muscular endurance vs. Hypertrophy	Glycaemia
Zaharieva et al. (27)	The effects of basal insulin suspension at the start of exercise on blood glucose levels during continuous vs. circuit-based exercise in individuals with type 1 diabetes on continuous subcutaneous insulin infusion	***N*** = Twelve adults **Age (years):** 32 **± 11** **BMI (kg/m**^**2**^**): 23.5** **Gender:** Mixed **♂** 6 ♀ 6 **VO**_**2max**_ **(ml·kg·min**^**−1**^**): 50.1 ± 13.7** **HbA1c (%): 7.0 ± 0.9** **Diabetes duration (years):−2–43**	**Intervention:** Acute **Exercise duration:** 40 min **Exercise Modality**: Mixed AE: Treadmill walk RE: Circuit **Exercise Intensity**: AE: 40–50% VO_2max_ RE: HIIE circuit training **Exercise Classification:** Circuit	Glycaemia
Reddy et al. (28)	Effect of aerobic and resistance exercise on glycemic control in adults with type 1 diabetes	***N*** **=** Ten adults **Age (years):** 33 ± 6 **BMI (kg/m**^**2**^**):** 24.4 ± 2.1 **Gender:** ♂ 4 ♀ 6 **Co-morbidities: -** **Exercise:** Physically active **VO**_**2max**_ **(ml·kg·min**^**−1**^**):** 46.8 ± 11.55 **HbA1c (%):** 7.4% ± 1% **Diabetes duration:** 18 ± 10	**Intervention:** Acute/Training (1 week) Two sessions of RE. Two sessions of AE. Control (no exercise) **Exercise duration**: 45 min **Exercise Modality**: RE: Whole body (Leg press, bench press, leg extension, leg flexion and seated row) AE: Treadmill running **Exercise Intensity**: RE:60–80% 1RM. 3 sets 8-12 repetitions AE: 60% VO_2max_ **Exercise Classification:** RE: Hypertrophy AE: MICE	Glycaemia

*N, number of participants; BMI, body mass index; HbA1c, Glycated haemoglobin; RE, Resistance exercise; AE, aerobic exercise; RM, repetition maximum; VO_2_, Volume of oxygen consumed; Reps, repetitions*.

## Morphological Adaptations—Skeletal Muscle Mass: Benefits Beyond Size

As an extremely heterogeneous tissue with immense plasticity, skeletal muscle influences locomotion, energy metabolism, thermodynamics, endocrine complexes, and cell signalling.

From a clinical perspective, the morphological adaptations induced by RET are associated with reduced rates of chronic inflammation (29–32), lowered global and central adiposity (31, 33), and a reduced risk of falls and fractures (34). Encouragingly, a recent article highlighted the credibility of RET in augmenting myocyte mitochondrial respiratory capacity and function (35). Considering both skeletal muscle insulin resistance and mitochondrial dysfunction are major features in the pathogenesis of T1D (36), interventions aimed at stimulating skeletal muscle growth are increasingly important in a diabetic milieu. The importance of preserved skeletal muscle mass is perhaps most evident in reference to age related muscle wasting or “sarcopenia.” This pathological derangement decreases the efficiency of several major metabolic processes, thereby accelerates the development risk of metabolic and mitochondrial abnormalities. Skeletal muscle mass is maintained when there is equilibrium between muscle protein synthesis (MPS) and muscle protein breakdown (37). A disturbance in this finite balance during which MPS exceeds the rate of protein breakdown induces skeletal muscle hypertrophy i.e., an increase in muscle size, whilst the opposite is said for atrophy i.e., a decrease in muscle size. The cellular mechanisms responsible for the initiation of growth involves the activation of various anabolic substrates including the phosphatidylinositol 3-kinase (P13K) signaling substrate mammalian target of rapamycin (mTOR) (38). mTOR is a serine/theronin kinase that integrates nutrient and metabolic stimuli to regulate cell growth and proliferation (39, 40). It's activation is dependent on sequential upregulations of P13K, phosphoinositide-dependent kinase (PDK)-1, 70-kDa ribosomal S6 kinase, and the Akt/PKB isoforms (41). Metabolically, mTOR increases glycolytic flux by activating the transcription and the translation of hypoxia inducible factor 1α (HIF1α) (42), whilst Akt facilitates the stimulation of glycogen synthase and formation by inhibiting glycogen synthase kinase-3 (31). HIF1α is an intermediate of several glycolytic and angiogenic genes including both vascular endothelial growth factor (VEGF) and erythropoietin (EPO) (43). VEGF triggers not only divisional but also phenotypical changes of the vascular endothelial cells to alter their migratory capacity and proliferation potential, whilst EPO stimulates erythropoiesis and therefore systemic O_2_ delivery. The hypertrophic potential of RE is not novel and its efficacy in substantially increasing rates of MPS in the many hours after exercise engagement are well-established (44). Biochemically, RE is known to evoke a transient increases in the phosphorylation of several of the aforementioned anabolic signalling molecules including mTOR (41), Akt/PKB (45), and 70-kDa ribosomal S6 kinase (45). Conversely, due to its inhibitory effect on several biochemical mediators of MPS, rates are minimal and/or unapparent following MICE. Encouragingly, recent research has demonstrated significant increases in upstream mediators of mTOR (growth hormone) in a volume dependent manner following acute RE in individuals with T1D (24). Since mTOR deregulation (46) and low CRF (47) are reported features of T1D, the biochemical cascades activated in response to appropriately designed RE are particularly noteworthy.

### Skeletal Muscle Strength and Fatigability

The functional significance of hypertrophied skeletal muscle is reflected by the accompanied increase in maximal strength and power (48). Muscular strength describes the amount of force a muscle can produce in a single effort, whilst muscular power describes the ability of the muscle to exert that force rapidly. The development of these two physical attributes extends to beyond enhancements in athletic performance. Strong, powerful muscles are necessary for the completion of simple, everyday tasks, which translate to greater independence and autonomy with aging, lower rates of all-cause mortality, enhanced fluidity of movement, and a decreased risk of injury (49). Beyond its pivotal role in locomotion, the clinical significance of hypertrophied muscle is also noted. The associations between reduced skeletal muscle strength and higher rates of clinical biomarkers of CVD are critically important in those with T1D, who are predisposed to a greater incidence of primary CVD throughout the lifespan (50). Indeed, research has highlighted a significantly lower muscle strength and fatigability in those with-vs.-without T1D (51). A physiological explanation for these declines may be evident within the muscle fibre type, since muscle fibre arrangement and/or type has a functional significance on the rate of force production and contraction velocity. Briefly, there exist two broad classifications of muscle fibres, Type 1, and Type II (the latter of which can be further subdivided to include Type IIa and IIx variations). However, it should be noted that whilst useful, these divisions are overly simplistic and do not reflect the reality of fibre type distribution—which includes a heterogenous mix within the skeletal muscle.

Structurally, there appears to be a reduced number of slow-twitch oxidative fibres (Type 1) coupled with an increased amount of the fast-twitch highly fatigable muscle fibres (Type II) in individuals with T1D. Combined, these increase the development of fatigue during maximal exercise and therefore necessitate earlier exercise cessation (52). Supporting these insights, previous work by Crowther and colleagues evidenced a significantly lower muscle pH at rest and at the end of exercise, indicating a greater reliance on glycolytic metabolism in the muscles of individuals with vs.-those-without T1D (53). Moreover, a significantly slower phosphocreatine (PCr) recovery time has been noted in adolescence with-vs.-without T1D, suggesting a reduced skeletal muscle oxidative profile with impaired recovery capacity (36). Whilst an altered skeletal muscle phenotype has been observed in T1D, the severity of these reductions appears to be magnified by the presence of diabetes-related complications. Recent work by Orlando and colleagues illustrated an impairment in the functionality of skeletal muscle in T1D patients with diabetic polyneuropathy (DPN) vs. those with complication free T1D (T1D) and healthy controls (C) (51). Findings revealed the DNP group had lower knee extensor muscle strength than both the T1D (−19%) and the C groups (−37.5%) as well as an elevated (22 and 45%) rate of lower body fatigability than the T1D and C groups, respectively. Critically, RE induces several neural adaptations, including disinhibition of inhibitory mechanisms, as well as improvements in both intra-and-inter-muscular co-ordination. This enhances the synchronisation of motor unit recruitment and firing capacity to facilitate greater levels of maximal force production. These adaptations are proportional to alteration in muscle morphology as seen by increases in muscle cross-sectional area (CSA) accompanied by increases in Type II muscle fibres and altered muscle architecture (fibre pennation) (37). A large part of this process is mediated by the central nervous system which acts as a highly complex control centre that facilitates movement at the molecular level (54).

## Metabolic Responses to Resistance Exercise

T1D is characterised by the progressive depletion and destruction of pancreatic β-cells accompanied with impaired glucagon-producing α-cell function. The resulting deficiency in endogenous insulin secretion manifests in chronic hyperglycemia, with the sequential need for a lifelong reliance on exogenous insulin therapy (55). On the other hand, hypoglycemia represents a perpetual clinical and conventional worry which independently constitutes a primary patient reported barrier to regular exercise participation (56). Therefore, a key consideration for those with T1D whom partake in any physical exercise is the maintenance of blood glucose (BG) control. However, whilst accounted for with little contemplation in regulatory metabolism, the attainment of this outcome requires a degree of diligent pre-planning in those with T1D, for whom variations in exercise modality, intensity, and duration require specific adjustments in therapeutic and feeding strategies in order to mitigate risk.

### Glycaemic Responses to Resistance Exercise

The control of BG during exercise involves the integrative co-ordination of two physiological systems (1) the autonomic nervous system (ANS) with particular emphasis on its sympathetic branch (SNS) and (2) the endocrine system (48). At a simplistic level, biochemical mediators released via these systems provide the feed-forward mechanisms that dictate glucose homeostasis. During moderate-to-high-intensity exercise, this feed-forward mechanism induces a rise in glucose concentrations which is primarily driven by hepatic glucose production (HGP). When glucose concentrations begin to fall as a result of sustained exercise efforts, the body produces a strong counter-regulatory response in an attempt to avoid hypoglycemia. This response is driven by the release of counter-regulatory hormones i.e., those that exert opposing actions to those of insulin. Recent work exploring the impact of single and multiple sets of RE on acute glycemic and glucoregulatory parameters has provided valuable endocrinological information in those with T1D (24). Researchers found significant increases in BG concentrations compared to resting conditions following one (+21%) and two (+29%) but not 3 sets of RE. The increases in BG were concurrent with a significant time^*^interaction effects in plasma catecholamines (noradrenaline and adrenaline). Indeed, peak catecholamine values were 28% greater under the 2nd set than the 1st set, and 17% greater under the 3rd set than the 2nd set. An increase in these two hormones parallels the idea that sympathoadrenergic activity can modulate HGP during exercise (57). Moreover, peak concentrations in growth hormone (GH) were observed during the 3rd (and final) set, which also agrees with work by Kraemer et al. who support the use of high volume session for maximising potential for hypertrophic outcomes (38). Furthermore, the considerable disturbance in acid-base balance observed during this protocol supports the “anaerobic” classification of RE that utilises predominately non-oxidative fuel metabolism via gluconeogenic pathways. Interestingly, despite the increasing hormonal and BG trajectory during the first two sets, the inclusion of a 3rd set produced BG concentrations similar to that of a non-exercise trial (24). It should also be noted that during recovery from 1 to 3 sets of RE, researchers reported no evidence of either exercise-induced hypoglycaemia or requirement for exogenous carbohydrates (CHO) (24). These findings oppose those commonly associated with AE, which typically reduces BG concentrations to a much greater extent (28, 58). Physiologically, the metabolic consequences of non-oxidative glycolytic activity likely contribute to the associated elevations in BG during the post exercise period, thereby offering protection against the onset of post-exercise hypoglycaemia. As a cautionary note, recent research has illustrated an increase in meal intake and after dinner snacking during the 24 h following both aerobic-and-resistance exercise sessions in order to prevent hypoglycaemia (28). Notably, findings highlighted significant reductions in post RE bolus insulin dosing requirements, which were also accompanied by less severe drops in plasma BG concentrations comparative to those recorded following AE (28). Accordingly, current recommendations are to reduce rapid acting insulin dosages alongside side a CHO rich pre-exercise meal (59, 60). This reductive strategy offsets the degree of hyperinsulinemia in people with T1D, which constitutes an inevitable outcome associated with exogenous insulin administration. Metabolically speaking, hyperinsulinemia exerts an inhibitory effect on fat utilisation during exercise, with the subsequently overreliance on CHO oxidation and therefore glucose dependency by the exercising skeletal muscle. Moreover, irrespective of interventional dose manipulations, insulin concentrations often increase during exercise (61). Mechanistically, this is likely due to an increased absorption rate from the subcutaneous tissue depot, together with a decreased clearance rate from at the systemic level. Combined the elevated degree of concentration of circulating of insulin propagates an increased risk of exercise-induced hypoglycaemia, which unsurprisingly prevails as a major barrier to exercise participation (56). Previous work has shown that postprandial exercise is associated with an increased risk of hypoglycemia if the pre-meal insulin dose is not reduced (62). Physiologically, this dose reduction strategy can augment the clearance rate of BG following the digestion of a CHO rich meal, thereby preserving BG concentrations ahead of exercise (62). The avoidance of in-exercise hyperinsulinemia has also been shown to replicate patterns of substrate oxidation and glucose regulation similar to a non-T1D individual (61). Moreover, a reduction in insulin mediated glucose uptake, in combination with elevations in catecholamine concentrations lessens the uptake and subsequent combustion of BG. Consequently, the major fuel substrates can be provided for both HGP and replenishment of muscle glycogen stores (63), such that a preservation effect is observed (64). Directionally, the cost of a slightly higher plasma glucose level before exercise translates to an improved glycemic profile during-and immediately-post-exercise (62).

It should be noted that when the exercise session is predominately reliant on non-oxidative fuel metabolism, post-exercise hyperglycaemia is common (23). The degree to which BG concentrations rise is dependent on several variables and can differ considerably both within and between individuals. Work by Turner and colleagues noted that when matched for volume, a comparable degree of post exercise hyperglycaemia is experienced in both low-and-high intensity morning RE sessions (26). Subsequent work highlighted the efficacy of administering an individualised dose of post-exercise rapid acting insulin in reducing the magnitude and severity of post-exercise hyperglycaemia, without causing early post-exercise hypoglycaemia in those with T1D (65). However, this protective effect waned thereafter, when prevalence rates of hypoglycaemia ensued during the later hours (65). Research has also demonstrated that the omission of pre-exercise bolus, but continuation of basal insulin dosages offers a protective effect against the occurrence of hypoglycaemia during and soon after a fasted bout of morning RE. A direct comparison between glycaemic responses to evening AE and RE in T1D revealed a decline in plasma glucose during a three-set evening RE session (albeit to a lesser extent than observed in the AE trial). Notably, there was also a tendency towards more frequent nocturnal hypoglycaemia following RE which warrants acknowledgment when considering the implementation of post exercise insulin dosing strategies to avoid acute hyperglycaemia. The differences in methodology between these two studies provides potential clues for contrasting glycaemic responses. Nevertheless, the authors concluded that in comparison to AE, RE may result in greater glycaemic stability both during and after exercise in those with T1D (20). Collectively, these data emphasise the need to account for exercise modality for the governance of appropriate insulin and feeding strategies around physical exercise in people with T1D.

### Relationships Between Skeletal Muscle, Obesity, and Insulin Resistance

The fact that skeletal muscle tissue is one of the largest organs in the human body, combined with the discovery that contracting skeletal muscle is a multifactorial control centre for the release of various genetic, cytokine and growth-related factors, supports the reasoning behind it's classification as an endocrine organ (31). Due to its mediatory role in promoting skeletal muscle vitality, integrity, and functionality, the potential of RE in influencing the progression or regression of several pathogenic disease states should not be overlooked. Although not historically a condition associated with obesity, there is an alarming percentage of individuals with T1D now categorised as either overweight or obese (66). Whilst intensive insulin therapy is often advocated to counteract poor glycaemic control, aggressive treatment can lead to weight gain, with the subsequent propensity to develop insulin resistance (IR). Worryingly, IR has become a recognised featured of T1D youth, accompanied by compromised exercise capacity and microvascular derangements (36). Moreover, in a study assessing the hemodynamic parameters to maximal exercise in patients with well-controlled T1D, researchers found IR to be inversely associated with cardiac output (67). IR occurs when an inadequate quantities of glucose transporter molecules (namely GLUT-4) migrate to the cell surface following insulin stimulation (31). The translocation site of GLUT-4 is the T-tubules found within the skeletal muscle, which highlights the potential of contracting skeletal muscle to uptake circulating glucose independently of insulin. Thus, whilst not novel, the “insulin-like” properties of skeletal muscle are extremely important in pathologies with metabolic abnormalities. As a fuel source, fat constitutes the most abundant energy pool in mammals and is stored in three major sites; adipose, skeletal muscle, and liver tissue. Each tissue contains depots of triacylglycerol molecules which are hydrolyzed to form fatty acids (FAs) and used as an energy source (31). However, an excessive accumulation of triacylglycerol leads to conditions of hyperlipidemia, with the subsequent propensity to develop IR (68). Skeletal muscle FA metabolism is acutely sensitive to physical exercise which causes substantial increases in intra and extra-cellular FA utilisation (31). Skeletal muscle is a leading consumer of FA from either lipoprotein-triacylglycerol or plasma non-essential fatty acids, and this consumption process rises exponentially during exercise. Finally, as a metabolically active tissue, skeletal muscle plays a pivotal role in maintaining body composition. Acutely, the prolonged window of increased MPS following RE induces elevations in thermogenesis and energy expenditure. Considering that skeletal muscle accounts for ~40% of total body mass and ~30% of basal metabolic rate (BMR) in adult humans (40), hypertrophied skeletal muscle may elevate resting muscle metabolism due to an increased ratio of skeletal muscle mass relative to body weight. Therefore, appropriately programmed exercise programmes that stimulate skeletal muscle hypertrophy may assist in weight reduction and/or management in those with T1D.

## Cardio-Vascular Responses to Resistance Exercise

For individuals with T1D, CVD is a major cause of morbidity and mortality and unsurprisingly constitutes a serious health concern that demands a prevention rather than treatment approach (69–71). Encouragingly, recent research has acknowledged the credibility of RE in reducing several CVD risk factors as well as improving various biomarkers of cardio-metabolic health (albeit it in a non-T1D cohorts) (72, 73). Although sparse, research has emphasised the efficacy of RET in improving traditional indices of CVD in people with T1D, including glycaemic and lipid related parameters (17). Furthermore, increases in heart rate (24, 26) and forearm reactive hyperaemia (25) have been reported following RE in individuals with T1D. Physiologically, the hypertrophic and angiogenic potential of RE is perhaps magnified in importance during circumstances where blood flow maybe compromised due to pathological abnormalities. Supporting this concept, researchers observed greater improvements in blood flow following lower limb RE vs. AE in individuals with T1D (25). However, these peripheral based improvements have thus far failed to correspond with improvements in cellular indices of vascular function. Indeed, a blunted circulating endothelial progenitor cell (cEPC) response has presented regardless of exercise modality, perhaps reflecting derangements in the microvascular domains. Although it should be noted that existing literature has thus far employed mostly MICE (74) and/or submaximal-lower limb RE (25).

## Skeletal Muscle: Important Micro-Vascular and Angiogenic Potentials

Prevalence rates of circulatory disease in people with T1D are extensive and at a population level constitute a leading cause of mortality (50). The pathophysiological mechanism responsible for the compromised circulatory system is most evident by the presence of abnormalities in the form and function of the blood vessels (50, 75, 76). Thus, whilst the identification of classical CVD risk factors provides valuable information as to an individual's current CVD vulnerability, they often fail to identify risk in its infancy and/or in those who appear to be asymptomatic. As such, physiological defects can go unrecognised until manifested as irreversible and/or severe acute complications within blood vessels including the presentation of endothelial dysfunction. Whilst the pathogenesis of macro-and-micro-vascular disease is multifactorial, the common recipient of injury is the vascular endothelium; a monolayer of cells that lines the blood vessels. Occupying a strategically important location between circulating blood and the surrounding tissues, the endothelium modulates the tone of the underlying vascular smooth muscle, maintains a non-adhesive luminal surface, mediates homeostasis, evokes cellular proliferation, and modulates inflammatory and immune mechanisms within the vascular wall (77). The functionality of the endothelium is mediated by a tightly regulated balance between the intermediates that induce cell damage i.e., pro-constrictive, pro-inflammatory, pro-thrombotic, and pro-hypertensive factors and the intermediates that support cell vitality i.e., pro-relaxation, anti-inflammatory, anti-thrombotic, and anti-hypertensive factors. Skeletal muscle hosts several sources of these vitality promoting intermediates, including the erythrocytes (which produce ATP and Nitric Oxide [NO]), the endothelial cells (which produce ATP, NO, and Prostacyclin [PGI_2_]), the skeletal muscle cells (which produce ATP, NO, and adenosine). Thus, during skeletal muscle contraction, there is an increased turnover of various vasodilatory factors that encourage blood flow and vascular repair processes. Indeed, the co-operative intimacy between skeletal muscle and its surrounding vasculature is made clear by the proximity of capillaries embedded in grooves indenting the sarcolemma and expanding the contact area between myocytes and endothelial cells (78). Muscle fibre mitochondria also appear to cluster along these groves, thereby shortening the travelling distance of O_2_ and various nutrients required for diffusion (78). The complex and intermate interaction between the biochemistry of the vascular endothelium, the vascular smooth muscle cells (VSMCs) and the skeletal muscle fibres emphasises the importance of endothelial vitality. Endothelial derangements proposedly contribute to the chronic under perfusion of skeletal muscle, resulting in suboptimal delivery and uptake of various nutrients, hormones and gaseous exchange. Indeed, these findings are somewhat consistent within the literature with research detailing higher rates of leg muscle deoxygenation (79), and altered red blood cell dynamics associated with the T1D milieu (80).

Mechanistically, microvascular abnormalities may be a result of lower skeletal muscle capillary density in individuals with T1D (81) which would agree with the aforementioned muscle fibre phenotype associated with T1D. Collectively, these attributes may contribute to a reduced circulatory ability to increase O_2_ delivery to exercising muscle. This may explain the lower rates of regular physical activity (56) as well as the earlier onset of fatigue observed in T1D during exercise testing where O_2_ delivery contributes to fatigue (82). Inefficient O_2_ and nutrient supply may also impact the vascular tone of skeletal muscle. Vascular responses to light exercise provide insight into the hierarchical regulation of muscle perfusion. With light exercise, vasomotion within the terminal arterioles appears to cease, and the number of perfused capillary increases without significant changes in total muscle blood flow. Thus, during MICE, there is less of an increase in shear stresses traversing over the vessel lumen than during higher intensity exercise. During higher intensity exercise, or following ischemia, under-perfused capillaries are recruited to ensure an increase muscle perfusion. Indeed, in people with T1D, 7 weeks of high-intensity sprint training has been shown reduced metabolic fluctuations and enhance muscle oxidative metabolism (83). Thus, as a major storage site of several nutrients including glucose, amino acids, and free fatty acids, skeletal muscle also plays an integral role in substrate provision during hypoxic or anaerobic conditions.

These insights suggest a credible role for exercise modalities that utilise compound movements performed at high intensity effort as a strategy for re-endothelisation. Perhaps, as a reflection of its capacity to induce ischemia and muscle microtrauma, exhaustive or eccentric exercise protocols including downhill running and RE appear to cause the greatest stress responses. The acute physiological “stress” experienced during these sessions augments the production and release of several mediators of re-endothelisation including stromal cell-derived factor 1 (SDF-1) (84) and VEGF (85). Upon activation, these proteins mediate progenitor cell mobilisation, proliferation, and migration (86). One of the most potent upstream mediators of EPC proliferation is VEGF, which is primarily known for its mediatory role in the (i) sprouting and (ii) intussusceptive splitting of capillary beds to form angiogenesis. Recent research has shown considerable elevations in VEGF concentrations within both skeletal muscle and the peripheral circulation following intense RE (87, 88). As such, the potential of exercise to induce local angiogenesis may assist in the prevention of ischemia within its surrounding tissue (43). Considering the decreased perfusion rates and increased atherosclerotic tendencies associated with T1D, the angiogenic potential of RE in this population is important.

## Osteopathic Responses to Resistance Exercise

The term “osteoporosis” describes inelasticity within the skeletal system when exposed to the habitual subjection of forces experienced during joint movement, locomotion, and loading. Bone mineral density (BMD) is used as a reference of bone mineralisation within bone tissue and is substantially influenced by endocrine activity. The endocrine and metabolic disturbances associated with T1D are hypothesised to contribute to higher rates of poor bone quality noted within the literature (89–91). Biochemically, T1D is associated with reductions in the anabolic agents of bone formation including IGF-1 and transforming growth factor beta 1 (TGFβ1), which initiate a potent stimulatory effect on the synthesis of bone-specific proteins and osteoblastic proliferation potential (92). Moreover, the downstream complications associated with hyperglycaemia including the direct inhibition of osteoblast activity, osmotic damage to osteoblasts, increased peroxisome proliferator-activated receptor activity and low microvascular proliferative capacity, may increase susceptibility to osteoporosis and fracture risk.

Physiologically, bones adapt to loading stimuli by upregulating osteoblast activity in the areas experiencing mechanical strain. With this in mind, the essence of RE may offer support against the poor bone quality sometimes observed in T1D. Supporting this concept, T1D centred paediatric research noted increases in both lean body mass (LBM) and BMD following 9-months of weight bearing activity (93). These physiological adaptations are mutually connected, since there seems to be a stoichiometric relationship between muscle and bone. Indeed, bone architecture depends critically on muscle CSA and tension development (94), whilst muscle immobilization, and/or detraining leads to bone mass loss (95). Moreover, exercises that require large rates of muscular force cause greater bone-related benefits than do isometric or low force exercises (94, 96–98). Conversely, extreme endurance and/or low load bearing activities may exasperate the degradation of bone quality and health (99, 100). Researchers have documented losses in bone mass during the competitive seasons in endurance cycling (100) whilst increases in bone mass are ascertained during the competitive seasons of gymnastics (95). Further research is needed to progress our understanding of the osteopathic responses to RE in individuals with T1D.

## Recommendations for Resistance Exercise in Type 1 Diabetes

**Figure 2:**
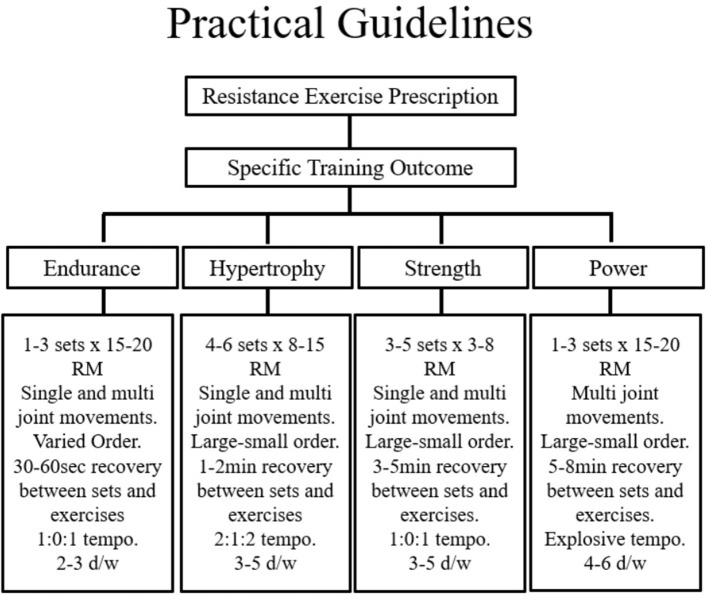
Proper programme design of resistance exercise for specific training outcomes incorporates the acute programme variables and key training principles. ECC, Eccentric; CON, Concentric; ISO, Isometric; Ex, exercise; Sec, seconds; RM, repetition maximum; Btn, Between; d/wk, Days per week. Figure adapted from Bird et al. [(104), Figure 2]. Springer Nature. Copyright © (Jan 1, 2005).

There are several acute manipulations which can substantially influence the outcome induced by RE. Indeed, DeLorme's classic work suggested that RET using low repetition/high resistance favoured adaptations for strength, power, and hypertrophy, whereas training with high repetition/low resistance increased muscular endurance and oxidative potential (101). From this, a repetition training continuum (102) or repetition maximum continuum (103) has been hypothesised such that the number of repetitions allowed by the resistance will result in very specific training adaptations. In order to enable a clear outline of program design, early work by Bird et al. (104) collated this information and created a visual representation of RE prescription. A modified version of this template that emphasises the points relevant to this review is included below (Figure 2).

Considering the focus of this review has emphasised the importance of skeletal muscle size the practical recommendations to follow will reflect the attainment of muscular hypertrophy. Skeletal muscle experiences the increases in size and strength when forced to contract at maximal or near-maximal efforts. Indeed, the rate of MPS is driven by the intracellular availability of amino acids—a factor which is influenced directly by the intensity and duration of muscle tension. With overall session intensity in mind, early research by Kramer and colleagues concluded that the combined effects of a higher volume, moderate intensity session with shorter rest periods provides a favourable hormonal milieu for promoting skeletal muscle growth (38). Thus, to maximise intensity, rests between exercises and sets should therefore be kept minimal (30–60 s) to maintain an elevated skeletal O_2_ demand that requires the use of predominately non-oxidative fuel metabolism.

Furthermore, the duration of muscular contraction significantly contributes to the phenotypic response. This process describes the “*tempo*” or time of the muscular contraction and is typically expressed in seconds. Moderate (2 s CON; 2 s ECC) and fast (1 s CON; 1 s ECC) velocities have been shown to maximise hormonal responses (105) and result in substantial metabolic cost (106). Classically the optimal repetition range for muscular hypertrophy is between 8 and 12. Initially, 2–3 sets may be enough for the untrained individual, however this should progress to >3 sets as with progressive overload. Moreover, programs that demand the simultaneous recruitment of large muscle groups traversing multiple joints in both the lower and upper body segments are advised. In this context, a program that emphasises compound movements towards the start of the session (when the muscle is in a “rested” state so can produce maximal force without fatigue), with isolated work grouped towards the end of a session is advised. In terms of exercise frequency, recommendations provided by the ADA (5) state that RE sessions aimed at skeletal muscle hypertrophy should be performed a minimum of twice but ideally thrice weekly.

Undertaking divergent exercise i.e., RE and MICE in close proximity may cause an “interference” effect on several of the acute molecular responses that orchestrate the adaptive phenotype. For example, activation of AMPK signalling in response to endurance exercise can block or dampen rates of MPS via its inhibitory effect on translation initiation and elongation (107). Moreover, the activation of AMPK is augmented in response to nutrient stress; particularly glycogen depletion; emphasising the need to acknowledge dietary considerations around exercise sessions. Thus, in an attempt to maximise MPS, individuals may wish to avoid both energy stress (increases in carbohydrate and amino acid feeding around exercise) and the concurrent practice of endurance and resistive exercise in close proximity (108).

Clearly exercise carries impactful glycaemic connotations for individuals with T1D. However, factors including the type, intensity and duration of exercise as well as the level of on-board exogenous insulin can considerably affect the glycemic responses that occur. Due to the pharmacological administration of exogenous insulin therapy, people with T1D frequently engage in exercise under markedly hyperinsulinemic conditions, thus are exposed to a greater risk of hypoglycaemia (62). As such, exercise often poses a glycaemic challenge which must be performed with a degree of contingency planning to both lifestyle and therapeutic strategies. It is prudent to suggest than insulin reductions and/or carbohydrate intake in the pre-and-post exercise periods are important features that need careful consideration with reference to BG values to appropriately manage glycemia. Exercise while fasting may produce a lesser decrease or indeed smaller increase in BG (65). It is advisory that BG is checked frequently in the hours pre, during and post exercise in order to govern safe and effective decisions regarding CHO feeding and insulin administration (5). RE induced hyperglycaemia risk can be abated by interspersing intense lifting efforts with lower intensity aerobic efforts (109) and/or combing RE (completed first) with AE (completed second) to optimize glycaemic stability (110). Further studies are needed to investigate the glycaemic impact of alterations in rapid-acting insulin strategies according to the type, intensity, and duration of the intended RE session.

## Future Research

Intuitively, the implementation of RE, a predominately glycolytic exercise that can be programmed to achieve global conditioning characterises a compelling therapeutic strategy that can be employed to improve several complications associated with T1D. However, despite the wider health merits associated with RE, a clear research emphasis has been placed on MICE with glycemia as its primary outcome.

In the existing RE specific research, program design has orientated around primarily hypertrophy or strength related outcomes, with less work investigating muscular endurance and/or power regimes for raising daily background levels of physical activity. In light of recent research emphasising the angiogenic potential of muscular endurance resistance exercise (MERE); during which healthy participants exercised to-or-near-to muscular failure (88), there presents a compelling rationale to employ these strategies in T1D by means of augmenting skeletal muscle angiogenesis. Indeed, the angiogenic and re-endothelialisation potential of MERE is particularly noted in this patient cohort, for whom circulatory complications are frequently observed (50). Moreover, considering the aforementioned alterations in the bioenergetics of skeletal muscle associated with T1D, the neuromuscular adaptations associated with moderate-to-heavy strength RE which incorporates a mixture of isometric and isokinetic movements i.e., greater motor unit recruitment, increased discharge rate modulation, larger muscle cross section area and changes in muscle architecture, are duly noted. Future research that explores the physiological responses to RE that *varies* in several of the aforementioned acute and chronic training variables would not only deepen our understanding from a research perspective but offer much clinical and patient relevance that may help develop healthcare guidelines specific to those with T1D.

## Conclusion

The importance of skeletal muscle strength and size extends far beyond it aesthetic and/or athletic performance value. Indeed, skeletal muscle plays an indispensable role in several key physiological processes including locomotion, hemodynamics, thermodynamics, cell signaling, and energy metabolism. Thus, the clinical merit of strategies that assist the maintenance and/or development of skeletal muscle are deserving of wider healthcare attention in patient populations. As a key determinant of muscle size and strength, the rate of MPS is critical for the development and/or preservation of skeletal muscle mass. This review has highlighted the potential of RE in upregulating the activity of several key biochemical and molecular signalling pathways that augment rates of MPS, including the PI3K/Akt/mTOR signalling pathway. Perhaps most importantly, the wider metabolic, vascular and respiratory roles of skeletal muscle each have particular relevance for individuals with T1D, whom are predisposed to greater pathological risk within these systems by virtue of the condition. Thus, the positive, adaptive health benefits associated with the hypertrophic and strength benefits accompanied with RE should be promoted as a necessary adjunct to standard diabetes care.

## Author Contributions

OlM: investigation and writing—original draft. OtM and RB: validation. OlM, RB, OtM, ME, RD, JP, and SB: writing—review and editing.

### Conflict of Interest Statement

OlM has received a Zienkiewcz Ph.D. research scholarship. OtM has received lecture fees from Medtronic, travel grants from Novo Nordisk A/S and EASD and research grants from Sêr Cymru II COFUND fellowship/European Union, Novo Nordisk A/S, Novo Nordisk Austria and Dexcom. ME has received a KESS2/European Social Fund scholarship. SB reports having received honoraria, teaching and research grants from the Abbott, Astra Zeneca, Boehringer Ingelheim, BMS, Diartis, Eli Lily and Company, GlaxoSmithKline, Johnson & Johnson, Merck Sharp & Dohme, Novartis, Novo Nordisk, Pfizer, Roche, Sanofi-Aventis, Schering-Plough, Servier and Takeda. RB reports having received honoraria, travel and educational grant support from, Beneo, Boehringer-Ingelheim, Eli Lily and Company, Novo Nordisk, Sanofi-Aventis. The remaining authors declare that the research was conducted in the absence of any commercial or financial relationships that could be construed as a potential conflict of interest.

## References

[1] Bohn B Herbst A Pfeifer M Krakow D Zimny S Kopp F. Impact of physical activity on glycemic control and prevalence of cardiovascular risk factors in adults with type 1 diabetes: a cross-sectional multicenter study of 18,028 patients. Diabetes Care. (2015) 38:1536–43. 10.2337/dc15-0030 26015557

[2] Riddell MC Perkins BA Type 1 diabetes and vigorous exercise: applications of exercise physiology to patient management. Can J Diabetes. (2006) 30:63–71. 10.1016/S1499-2671(06)01010-0

[3] Granata C Jamnick NA Bishop DJ Principles of exercise prescription, and how they influence exercise-induced changes of transcription factors and other regulators of mitochondrial biogenesis. Sport Med. (2018) 48:1541–59. 10.1007/s40279-018-0933-1 29675670

[4] Moy CS Songer TJ LaPorte RE Dorman JS Kriska AM Orchard TJ. Insulin-dependent diabetes mellitus, physical activity, and death. Am J Epidemiol. (1993) 137:74–81. 10.1093/oxfordjournals.aje.a116604 8434575

[5] Colberg SR Sigal RJ Yardley JE Riddell MC Dunstan DW Dempsey PC. Physical activity/exercise and diabetes: a position statement of the American Diabetes Association. Diabetes Care. (2016) 39:2065–79. 10.2337/dc16-1728 27926890 PMC6908414

[6] Kriska AM LaPorte RE Patrick SL Kuller LH Orchard TJ. The association of physical activity and diabetic complications in individuals with insulin-dependent diabetes mellitus: the Epidemiology of Diabetes Complications Study–VII. J Clin Epidemiol. (1991) 44:1207–14. 10.1016/0895-4356(91)90153-Z 1941015

[7] LaPorte RE Dorman JS Tajima N Cruickshanks KJ Orchard TJ Cavender DE. Pittsburgh insulin-dependent diabetes mellitus morbidity and mortality study: physical activity and diabetic complications. Pediatrics. (1986) 78:1027–33. 3786027

[8] Edmunds S Roche D Stratton G Wallymahmed K Glenn SM. Physical activity and psychological well-being in children with Type 1 diabetes. Psychol Health Med. (2007) 12:353–63. 10.1080/13548500600975446 17510906

[9] Imayama I Plotnikoff RC Courneya KS Johnson JA. Determinants of quality of life in adults with type 1 and type 2 diabetes. Health Qual Life Outcomes. (2011) 9:115. 10.1186/1477-7525-9-115 22182307 PMC3258220

[10] Codella R Terruzzi I Luzi L. Why should people with type 1 diabetes exercise regularly? Acta Diabetol. (2017) 54:615–30. 10.1007/s00592-017-0978-x 28289908

[11] Garber CE Blissmer B Deschenes MR Franklin BA Lamonte MJ Lee IM. Quantity and quality of exercise for developing and maintaining cardiorespiratory, musculoskeletal, and neuromotor fitness in apparently healthy adults. Med Sci Sport Exerc. (2011) 43:1334–59. 10.1249/MSS.0b013e318213fefb 21694556

[12] Baldi JC Cassuto NA Foxx-Lupo WT Wheatley CM Snyder EM. Glycemic status affects cardiopulmonary exercise response in athletes with type I diabetes. Med Sci Sport Exerc. (2010) 42:1454–9. 10.1249/MSS.0b013e3181d1fdb3 20139786

[13] Moser O Eckstein ML McCarthy O Deere R Bain SC Haahr HL. Poor glycaemic control is associated with reduced exercise performance and oxygen economy during cardio-pulmonary exercise testing in people with type 1 diabetes. Diabetol Metab Syndr. (2017) 9:93. 10.1186/s13098-017-0294-1 29201153 PMC5697085

[14] Moser O Eckstein ML McCarthy O Deere R Bain SC Haahr HL. Heart rate dynamics during cardio-pulmonary exercise testing are associated with glycemic control in individuals with type 1 diabetes. PLoS ONE. (2018) 13:e0194750. 10.1371/journal.pone.0194750 29608593 PMC5880363

[15] Parolin ML Chesley A Matsos MP Spriet LL Jones NL Heigenhauser GJ. Regulation of skeletal muscle glycogen phosphorylase and PDH during maximal intermittent exercise. Am. J. Physiol. (1999) 277:E890–900. 10.1152/ajpendo.1999.277.5.E890 10567017

[16] Volaklis KA Tokmakidis SP. Resistance exercise training in patients with heart failure. Sports Med. (2005) 35:1085–103. 10.2165/00007256-200535120-00006 16336010

[17] Durak EP Jovanovic-Peterson L Peterson CM. Randomized crossover study of effect of resistance training on glycemic control, muscular strength, and cholesterol in type I diabetic men. Diabetes Care. (1990) 13:1039–43. 10.2337/diacare.13.10.1039 2209300

[18] Jimenez C Santiago M Sitler M Boden G Homko C. Insulin-sensitivity response to a single bout of resistive exercise in type 1 diabetes. J Sport Rehabil. (2009) 18:564–71. 10.1123/jsr.18.4.564 20108856

[19] D'hooge R Hellinckx T Van Laethem C Stegen S De Schepper J Van Aken S. Influence of combined aerobic and resistance training on metabolic control, cardiovascular fitness and quality of life in adolescents with type 1 diabetes: a randomized controlled trial. Clin Rehabil. (2011) 25:349–59. 10.1177/0269215510386254 21112904

[20] Yardley JE Kenny GP Perkins BA Riddell MC Balaa N Malcolm J Resistance vs. aerobic exercise: acute effects on glycemia in type 1 diabetes - ProQuest. Diabetes Care. (2013) 36:537–42. 10.2337/dc12-0963 23172972 PMC3579339

[21] Yardley JE Kenny GP Perkins BA Riddell MC Malcolm J Boulay P Effects of performing resistance exercise before vs. after aerobic exercise on glycemia in type 1 diabetes. Diabetes Care. (2012) 35:669–75. 10.2337/dc11-1844 22374639 PMC3308306

[22] Silveira AP Bentes CM Costa PB Simão R Silva FC Silva RP. Acute effects of different intensities of resistance training on glycemic fluctuations in patients with type 1 diabetes mellitus. Res Sport Med. (2014) 22:75–87. 10.1080/15438627.2013.852096 24392773

[23] Turner D Luzio S Kilduff LP Gray BJ Dunseath G Bain SC. Reductions in resistance exercise-induced hyperglycaemic episodes are associated with circulating interleukin-6 in Type 1 diabetes. Diabet Med. (2014) 31:1009–13. 10.1111/dme.12462 24702172

[24] Turner D Luzio S Gray BJ Dunseath G Rees ED Kilduff LP. Impact of single and multiple sets of resistance exercise in type 1 diabetes. Scand J Med Sci Sports. (2015) 25:e99–e109. 10.1111/sms.12202 24646137

[25] Waclawovsky G Umpierre D Figueira FR De Lima ES Alegretti AP Schneider L. Exercise on progenitor cells in healthy subjects and patients with type 1 diabetes. Am Coll Sport Med. (2015) 48:190–9. 10.1249/MSS.0000000000000764 26312614

[26] Turner D Gray BJ Luzio S Dunseath G Bain SC Hanley S. Similar magnitude of post-exercise hyperglycemia despite manipulating resistance exercise intensity in type 1 diabetes individuals. Scand J Med Sci Sports. (2016) 26:404–12. 10.1111/sms.12472 25919405

[27] Zaharieva D Yavelberg L Jamnik V Cinar A Turksoy K Riddell MC The effects of basal insulin suspension at the start of exercise on blood glucose levels during continuous vs. circuit-based exercise in individuals with type 1 diabetes on continuous subcutaneous insulin infusion. Diabetes Technol Ther. (2017) 19:370–8. 10.1089/dia.2017.0010 28613947 PMC5510047

[28] Reddy R Wittenberg A Castle JR El Youssef J Winters-Stone K Gillingham M Effect of aerobic and resistance exercise on glycemic control in adults with type 1 diabetes. Can J Diabetes. (2018) 43:406–14.e1. 10.1016/j.jcjd.2018.08.193 30414785 PMC6591112

[29] Mccall GE Byrnes WC Dickinson A Pattany PM Fleck SJ. Muscle fiber hypertrophy, hyperplasia, and capillary density in college men after resistance training. J Appl Physiol. (1996) 81:2004–12. 10.1152/jappl.1996.81.5.2004 8941522

[30] Kemi OJ Haram PM Loennechen JP Osnes JB Skomedal T Wisløff U. Moderate vs. high exercise intensity: differential effects on aerobic fitness, cardiomyocyte contractility, and endothelial function. Cardiovasc Res. (2005) 67:161–72. 10.1016/j.cardiores.2005.03.010 15949480

[31] Wagenmakers AJM The Biochemical Basis of the Health Effects of Exercise. London: Portland Press (2006).

[32] Morici G Zangla D Santoro A Pelosi E Petrucci E Gioia M. Supramaximal exercise mobilizes hematopoietic progenitors and reticulocytes in athletes. AJP Regul Integr Comp Physiol. (2005) 289:R1496–R1503. 10.1152/ajpregu.00338.2005 16020520

[33] Reid CM Yeater RA Ullrich IH. Weight training and strength, cardiorespiratory functioning and body composition of men. Br J Sports Med. (1987) 21:40–4. 10.1136/bjsm.21.1.40 3580729 PMC1478605

[34] Siegrist M. Role of physical activity in the prevention of osteoporosis. Med Monatsschr Pharm. (2008) 31:259–264. 18808074

[35] Porter C Reidy PT Bhattarai N Sidossis LS Rasmussen BB. Resistance exercise training alters mitochondrial function in human skeletal muscle. Med Sci Sports Exerc. (2015) 47:1922–31. 10.1249/MSS.0000000000000605 25539479 PMC4478283

[36] Cree-Green M Newcomer BR Brown MS Baumgartner AD Bergman B Drew B. Delayed skeletal muscle mitochondrial ADP recovery in youth with type 1 diabetes relates to muscle insulin resistance. Diabetes. (2015) 64:383–92. 10.2337/db14-0765 25157095 PMC4303961

[37] Cardinale M Newton RU Nosaka K Strength and Conditioning : Biological Principles and Practical Applications. Chichester: John Wiley & Sons (2011).

[38] Kraemer WJ Gordon SE Fleck SJ Marchitelli LJ Mello R Dziados JE. Endogenous anabolic hormonal and growth factor responses to heavy resistance exercise in males and females. Int J Sports Med. (1991) 12:228–35. 10.1055/s-2007-1024673 1860749

[39] Cho H Mu J Kim JK Thorvaldsen JL Chu Q Crenshaw EB III Insulin resistance and a diabetes mellitus-like syndrome in mice lacking the protein kinase Akt2 (PKBbeta). Science. (2001) 292:1728–31. 10.1126/science.292.5522.1728 11387480

[40] Egan B Zierath JR. Exercise metabolism and the molecular regulation of skeletal muscle adaptation. Cell Metab. (2013) 17:162–84. 10.1016/j.cmet.2012.12.012 23395166

[41] Parkington JD Siebert AP LeBrasseur NK Fielding RA. Differential activation of mTOR signaling by contractile activity in skeletal muscle. Am J Physiol Integr Comp Physiol. (2003) 285:R1086–R1090. 10.1152/ajpregu.00324.2003 12881204

[42] Ribeiro F Ribeiro IP Gonçalves AC Alves AJ Melo E Fernandes R. Effects of resistance exercise on endothelial progenitor cell mobilization in women. Sci Rep. (2017) 7:17880. 10.1038/s41598-017-18156-6 29259281 PMC5736626

[43] Golbidi S Laher I. Exercise and the cardiovascular system. Cardiol Res Pract. (2012) 2012:1–15. 10.1155/2012/210852 22701195 PMC3371347

[44] Chesley A MacDougall JD Tarnopolsky MA Atkinson SA Smith K. Changes in human muscle protein synthesis after resistance exercise. J Appl Physiol. (1992) 73:1383–8. 10.1152/jappl.1992.73.4.1383 1280254

[45] Nader GA Esser KA. Intracellular signaling specificity in skeletal muscle in response to different modes of exercise. J Appl Physiol. (2001) 90:1936–42. 10.1152/jappl.2001.90.5.1936 11299288

[46] Gordon CS Serino AS Krause MP Campbell JE Cafarelli E Adegoke OA. Impaired growth and force production in skeletal muscles of young partially pancreatectomized rats: a model of adolescent type 1 diabetic myopathy? PLoS ONE. (2010) 5:e14032. 10.1371/journal.pone.0014032 21103335 PMC2984438

[47] Williams BK Guelfi KJ Jones TW Davis EA. Lower cardiorespiratory fitness in children with Type 1 diabetes. Diabet Med. (2011) 28:1005–7. 10.1111/j.1464-5491.2011.03271.x 21749445

[48] Brooks GA Fahey TD Baldwin KM Exercise Physiology : Human Bioenergetics and Its Applications. New York, NY: McGraw-Hill (2005).

[49] Ruiz JR Sui X Lobelo F Morrow JR Jackson AW Sjöström M. Association between muscular strength and mortality in men: prospective cohort study. BMJ. (2008) 337:a439. 10.1136/bmj.a439 18595904 PMC2453303

[50] Livingstone SJ Looker HC Hothersall EJ Wild SH Lindsay RS Chalmers J. Risk of cardiovascular disease and total mortality in adults with type 1 diabetes: Scottish Registry Linkage Study. PLoS Med. (2012) 9:e1001321. 10.1371/journal.pmed.1001321 23055834 PMC3462745

[51] Orlando G Balducci S Bazzucchi I Pugliese G Sacchetti M. The impact of type 1 diabetes and diabetic polyneuropathy on muscle strength and fatigability. Acta Diabetol. (2017) 54:543–50. 10.1007/s00592-017-0979-9 28285381

[52] Andreassen CS Jensen JM Jakobsen J Ulhøj BP Andersen H. Striated muscle fiber size, composition, and capillary density in diabetes in relation to neuropathy and muscle strength. J Diabetes. (2014) 6:462–71. 10.1111/1753-0407.12124 24397623

[53] Crowther GJ Milstein JM Jubrias SA Kushmerick MJ Gronka RK Conley KE. Altered energetic properties in skeletal muscle of men with well-controlled insulin-dependent (type 1) diabetes. Am J Physiol Metab. (2003) 284:E655–E662. 10.1152/ajpendo.00343.2002 12626321

[54] Carroll TJ Riek S Carson RG. Neural adaptations to resistance training. Sport Med. (2001) 31:829–40. 10.2165/00007256-200131120-00001 11665911

[55] Atkinson MA Eisenbarth GS. Type 1 diabetes: new perspectives on disease pathogenesis and treatment. Lancet. (2001) 358:221–9. 10.1016/S0140-6736(01)05415-0 11476858

[56] Brazeau A-S Rabasa-Lhoret R Strychar I Mircescu H. Barriers to physical activity among patients with type 1 diabetes. Diabetes Care. (2008) 31:2108–9. 10.2337/dc08-0720 18689694 PMC2571055

[57] Hargreaves M Spriet LL Exercise Metabolism. (2006). Available online at: https://uk.humankinetics.com/products/exercise-metabolism-2nd-edition (accessed August 12, 2018).

[58] Yardley JE The Acute Effects of Aerobic and Resistance Exercise on Blood Glucose Levels in Type 1 Diabetes. (2011). Available online at: https://ruor.uottawa.ca/bitstream/10393/20031/5/Yardley_Jane_Elizabeth_2011_thesis.pdf (accessed January 8, 2018).

[59] West DJ Stephens JW Bain SC Kilduff LP Luzio S Still R. A combined insulin reduction and carbohydrate feeding strategy 30 min before running best preserves blood glucose concentration after exercise through improved fuel oxidation in type 1 diabetes mellitus. J Sports Sci. (2011) 29:279–89. 10.1080/02640414.2010.531753 21154013

[60] Campbell MD Walker M Trenell MI Luzio S Dunseath G Tuner D. Metabolic implications when employing heavy pre- and post-exercise rapid-acting insulin reductions to prevent hypoglycaemia in type 1 diabetes patients: a randomised clinical trial. PLoS ONE. (2014) 9:e97143. 10.1371/journal.pone.0097143 24858952 PMC4032262

[61] Chokkalingam K Tsintzas K Norton L Jewell K Macdonald IA Mansell PI. Exercise under hyperinsulinaemic conditions increases whole-body glucose disposal without affecting muscle glycogen utilisation in type 1 diabetes. Diabetologia. (2007) 50:414–21. 10.1007/s00125-006-0520-0 17119916

[62] Rabasa-Lhoret R Bourque J Ducros F Chiasson JL. Guidelines for premeal insulin dose reduction for postprandial exercise of different intensities and durations in type 1 diabetic subjects treated intensively with a basal-bolus insulin regimen (ultralente-lispro). Diabetes Care. (2001) 24:625–30. 10.2337/diacare.24.4.625 11315820

[63] Steppel JH Horton ES. Exercise in the management of type 1 diabetes mellitus. Rev Endocr Metab Disord. (2003) 4:355–60. 10.1023/A:1027302112655 14618020

[64] Cryer PE Davis SN Shamoon H. Hypoglycemia in diabetes. Diabetes Care. (2003) 26:1902–12. 10.2337/diacare.26.6.1902 12766131

[65] Turner D Luzio S Gray BJ Bain SC Hanley S Richards A. Algorithm that delivers an individualized rapid-acting insulin dose after morning resistance exercise counters post-exercise hyperglycaemia in people with Type 1 diabetes. Diabet Med. (2016) 33:506–10. 10.1111/dme.12870 26220149

[66] Conway B Miller RG Costacou T Fried L Kelsey S Evans RW. Temporal patterns in overweight and obesity in Type 1 diabetes. Diabet Med. (2010) 27:398–404. 10.1111/j.1464-5491.2010.02956.x 20536510 PMC3129711

[67] Niedzwiecki P Naskret D Pilacinski S Pempera M Uruska A Adamska A. The higher the insulin resistance the lower the cardiac output in men with type 1 diabetes during the maximal exercise test. Metab Syndr Relat Disord. (2017) 15:252–257. 10.1089/met.2017.0007 28394194

[68] Koves TR Ussher JR Noland RC Slentz D Mosedale M Ilkayeva O. Mitochondrial overload and incomplete fatty acid oxidation contribute to skeletal muscle insulin resistance. Cell Metab. (2008) 7:45–56. 10.1016/j.cmet.2007.10.013 18177724

[69] Kannel WB McGee DL. Diabetes and cardiovascular disease. JAMA. (1979) 241:2035. 10.1001/jama.1979.03290450033020 430798

[70] Vistisen D Andersen GS Hansen CS Hulman A Henriksen JE Bech-Nielsen H Prediction of first cardiovascular disease event in type 1 diabetes: the steno T1 risk engine. Circulation. (2016) 133:1058–66. 10.1161/CIRCULATIONAHA.115.018844 26888765

[71] Rueda SF Fernández C Nicolau J Ricart MJ Esmatjes E. Prevalence of cardiovascular risk factors in patients with type 1 diabetes in end-stage renal disease: changes in the trend from 1999 to 2006. J Diabetes Complications. (2009) 23:317–22. 10.1016/j.jdiacomp.2008.01.003 18358752

[72] Yang Z Scott CA Mao C Tang J Farmer AJ Resistance exercise vs. aerobic exercise for type 2 diabetes: a systematic review and meta-analysis. Sport Med. (2014) 44:487–99. 10.1007/s40279-013-0128-8 24297743

[73] Shiroma EJ Cook NR Manson JE Moorthy MV Buring JE Rimm EB. Strength training and the risk of type 2 diabetes and cardiovascular disease. Med Sci Sport Exerc. (2017) 49:40–6. 10.1249/MSS.0000000000001063 27580152 PMC5161704

[74] West DJ Campbell MD Gonzalez JT Walker M Stevenson EJ Ahmed FW. The inflammation, vascular repair and injury responses to exercise in fit males with and without Type 1 diabetes: an observational study. Cardiovasc Diabetol. (2015) 14:71. 10.1186/s12933-015-0235-y 26044827 PMC4460651

[75] Loomans CJM de Koning EJP Staal FJT Rookmaaker MB Verseyden C de Boer HC. Endothelial progenitor cell dysfunction: a novel concept in the pathogenesis of vascular complications of type 1 diabetes. Diabetes. (2004) 53:195–9. 10.2337/diabetes.53.1.195 14693715

[76] Gordin D Wadén J Forsblom C Thorn LM Rosengård-Bärlund M Heikkilä O. Arterial stiffness and vascular complications in patients with type 1 diabetes: the Finnish Diabetic Nephropathy (FinnDiane) Study. Ann Med. (2012) 44:196–204. 10.3109/07853890.2010.530681 21047152

[77] Rubanyi GM. The role of endothelium in cardiovascular homeostasis and diseases. J Cardiovasc Pharmacol. (1993) 22 (Suppl 4):S1–14. 10.1097/00005344-199322004-00002 7523767

[78] Kusters YHAM Barrett EJ. Muscle microvasculature's structural and functional specializations facilitate muscle metabolism. Am J Physiol Endocrinol Metab. (2016) 310:E379–87. 10.1152/ajpendo.00443.2015 26714849 PMC4888529

[79] Peltonen JE Koponen AS Pullinen K Hägglund H Aho JM Kyröläinen H. Alveolar gas exchange and tissue deoxygenation during exercise in type 1 diabetes patients and healthy controls. Respir Physiol Neurobiol. (2012) 181:267–76. 10.1016/j.resp.2012.04.002 22538274

[80] Kindig CA Sexton WL Fedde MR Poole DC. Skeletal muscle microcirculatory structure and hemodynamics in diabetes. Respir Physiol. (1998) 111:163–75. 10.1016/S0034-5687(97)00122-9 9574868

[81] Krause MP Riddell MC Hawke TJ. Effects of type 1 diabetes mellitus on skeletal muscle: clinical observations and physiological mechanisms. Pediatr Diabetes. (2011) 12(4 Pt 1):345–64. 10.1111/j.1399-5448.2010.00699.x 20860561

[82] Stubbe B Schipf S Schäper C Felix SB Steveling A Nauck M. The influence of type 1 diabetes mellitus on pulmonary function and exercise capacity – results from the study of health in pomerania (SHIP). Exp Clin Endocrinol Diabetes. (2016) 125:64–9. 10.1055/s-0042-112219 27701716

[83] Harmer AR Chisholm DJ McKenna MJ Hunter SK Ruell PA Naylor JM. Sprint training increases muscle oxidative metabolism during high-intensity exercise in patients with type 1 diabetes. Diabetes Care. (2008) 31:2097–102. 10.2337/dc08-0329 18716051 PMC2571053

[84] Stellos K Gawaz M. Platelets and stromal cell-derived factor-1 in progenitor cell recruitment. Semin Thromb Hemost. (2007) 33:159–64. 10.1055/s-2007-969029 17340464

[85] Asahara T Takahashi T Masuda H Kalka C Chen D Iwaguro H. VEGF contributes to postnatal neovascularization by mobilizing bone marrow-derived endothelial progenitor cells. EMBO J. (1999) 18:3964–72. 10.1093/emboj/18.14.3964 10406801 PMC1171472

[86] Sen S McDonald SP Coates PTH Bonder CS. Endothelial progenitor cells: novel biomarker and promising cell therapy for cardiovascular disease. Clin Sci. (2011) 120:263–83. 10.1042/CS20100429 21143202

[87] Gavin TP Drew JL Kubik CJ Pofahl WE Hickner RC. Acute resistance exercise increases skeletal muscle angiogenic growth factor expression. Acta Physiol. (2007) 191:139–46. 10.1111/j.1748-1716.2007.01723.x 17565567

[88] Ross MD Wekesa AL Phelan JP Harrison M. Resistance exercise increases endothelial progenitor cells and angiogenic factors. Med Sci Sports Exerc. (2014) 46:16–23. 10.1249/MSS.0b013e3182a142da 24346188

[89] Tonoli C Heyman E Buyse L Roelands B Piacentini MF Bailey S. Neurotrophins and cognitive functions in T1D compared with healthy controls: effects of a high-intensity exercise. Appl Physiol Nutr Metab. (2015) 40:20–7. 10.1139/apnm-2014-0098 25525862

[90] Maddaloni E D'Eon S Hastings S Tinsley LJ Napoli N Khamaisi M. Bone health in subjects with type 1 diabetes for more than 50 years. Acta Diabetol. (2017) 54:479–88. 10.1007/s00592-017-0973-2 28236093 PMC5406751

[91] Parajuli A Liu C Li W Gu X Lai X Pei S. Bone's responses to mechanical loading are impaired in type 1 diabetes. Bone. (2015) 81:152–60. 10.1016/j.bone.2015.07.012 26183251 PMC4640966

[92] de Souza KS Ururahy MA da Costa Oliveira YM Loureiro MB da Silva HP Bortolin RH. Low bone mineral density in patients with type 1 diabetes: association with reduced expression of *IGF1, IGF1R* and *TGF B 1* in peripheral blood mononuclear cells. Diabetes Metab Res Rev. (2016) 32:589–95. 10.1002/dmrr.2772 26663878

[93] Maggio AB Rizzoli RR Marchand LM Ferrari S Beghetti M Farpour-Lambert NJ. Physical activity increases bone mineral density in children with type 1 diabetes. Med Sci Sport Exerc. (2012) 44:1206–11. 10.1249/MSS.0b013e3182496a25 22246217

[94] Rittweger J Beller G Ehrig J Jung C Koch U Ramolla J. Bone-muscle strength indices for the human lower leg. Bone. (2000) 27:319–26. 10.1016/S8756-3282(00)00327-6 10913929

[95] Snow CM Williams DP LaRiviere J Fuchs RK Robinson TL. Bone gains and losses follow seasonal training and detraining in gymnasts. Calcif Tissue Int. (2001) 69:7–12. 10.1007/s00223-001-0014-5 11685427

[96] Gibbs JC Craven BC Moore C Thabane L Adachi JD Giangregorio LM. Muscle density and bone quality of the distal lower extremity among individuals with chronic spinal cord injury. Top Spinal Cord Inj Rehabil. (2015) 21:282–93. 10.1310/sci2104-282 26689693 PMC4750813

[97] Lang TF. The bone-muscle relationship in men and women. J Osteoporos. (2011) 2011:702735. 10.4061/2011/702735 22007336 PMC3189615

[98] Burt LA Ducher G Naughton GA Courteix D Greene DA. Gymnastics participation is associated with skeletal benefits in the distal forearm: a 6-month study using peripheral Quantitative Computed Tomography. J Musculoskelet Neuronal Interact. (2013) 13:395–404. 24292609

[99] Folscher L-L Grant CC Fletcher L Janse van Rensberg DC. Ultra-marathon athletes at risk for the female athlete triad. Sport Med Open. (2015) 1:29. 10.1186/s40798-015-0027-7 26380807 PMC4564455

[100] Barry DW Kohrt WM. BMD decreases over the course of a year in competitive male cyclists. J Bone Miner Res. (2008) 23:484–91. 10.1359/jbmr.071203 18072875

[101] Todd JS Shurley JP Todd TC. Thomas L. DeLorme and the science of progressive resistance exercise. J Strength Cond Res. (2012) 26:2913–23. 10.1519/JSC.0b013e31825adcb4 22592167

[102] Anderson T Kearney JT. Effects of three resistance training programs on muscular strength and absolute and relative endurance. Res Q Exerc Sport. (1982) 53:1–7. 10.1080/02701367.1982.10605218 7079558

[103] Fleck SJ Kraemer WJ. Resistance training: basic principles (Part 1 of 4). Phys Sportsmed. (1988) 16:160–71. 10.1080/00913847.1988.11709461 27404835

[104] Bird SP Tarpenning KM Marino FE. Designing resistance training programmes to enhance muscular fitness. Sport Med. (2005) 35:841–51. 10.2165/00007256-200535100-00002 16180944

[105] Kraemer RR Kilgore JL Kraemer GR Castracane VD. Growth hormone, IGF-I, and testosterone responses to resistive exercise. Med Sci Sports Exerc. (1992) 24:1346–1352. 10.1249/00005768-199212000-00007 1470017

[106] Hunter GR Seelhorst D Snyder S. Comparison of metabolic and heart rate responses to super slow vs. traditional resistance training. J strength Cond Res. (2003) 17:76–81. 10.1519/1533-4287(2003)017<0076:COMAHR>2.0.CO;2 12580660

[107] Coffey VG Pilegaard H Garnham AP O'Brien BJ Hawley JA. Consecutive bouts of diverse contractile activity alter acute responses in human skeletal muscle. J Appl Physiol. (2009) 106:1187–97. 10.1152/japplphysiol.91221.2008 19164772

[108] Spurway N Wackerhage H British Association of Sport and Exercise Sciences. Genetics and Molecular Biology of Muscle Adaptation. Amsterdam: Churchill Livingstone/Elsevier (2006).

[109] Guelfi KJ Jones TW Fournier PA. The decline in blood glucose levels is less with intermittent high-intensity compared with moderate exercise in individuals with type 1 diabetes. Diabetes Care. (2005) 28:1289–94. 10.2337/diacare.28.6.1289 15920041

[110] Yardley JE Sigal RJ Riddell MC Perkins BA Kenny GP Performing resistance exercise before vs. vs. after aerobic exercise influences growth hormone secretion in type 1 diabetes. Appl Physiol Nutr. (2014) 39:262–5. 10.1139/apnm-2013-0329 24476484

